# Bilateral Pneumothorax Associated With Lung and Pleural Metastases of Breast Cancer

**DOI:** 10.1155/DTE.7.69

**Published:** 2001

**Authors:** Terumasa Kurihara, Osamu Kawashima, Keiichi Endo, Yuichi Iino, Susumu Ishikawa, Yasuo Morishita

**Affiliations:** 1 Department of Surgery National Nishi-gunma Hospital 2854 Kanai Shibukawa Gunma 377-8511 Japan; 2 Department of Emergency and Critical Care Medicine Gunma University School of Medicine 3-39-15 Showa-machi Maebashi Gunma 371-8511 Japan; 3 Second Department of Surgery Gunma University School of Medicine 3-39-15 Showa-machi Maebashi Gunma 371-8511 Japan

## Abstract

A rare case of bilateral pneumothorax in a 54-year-old woman with advanced breast cancer associated with lung and pleural metastases is presented. The patient was admitted to our hospital complaining of unexpected severe dyspnea. A chest X-ray showed bilateral pneumothorax associated with multiple lung metastases and pleural effusions, followed by immediate pleural drainage. Although air leak and effusions of the right lung were well controlled by the conservative management, massive air leaks of the left lung had continued for 40 days. Because of patient's poor general status a surgical closure of the leaking site was selected using video-assisted thoracoscopic surgery techniques. Thoracoscopy revealed a ruptured bulla in the lower lobe (S^6^), thus, followed by a successful bullectomy with a stapling device. We speculate that multiple pleural metastasis may disturb the normal repair mechanism of the lung tissue and cause prolonged persistent air leaks.


*Diagnostic and Therapeutic Endoscopy*, Vol. 7, pp. 69–73
Reprints available directly from the publisher
Photocopying permitted by license only

© 2001 OPA (Overseas Publishers Association) N.V.
Published by license under
the Harwood Academic Publishers imprint,
part of Gordon and Breach Publishing,
a member of the Taylor & Francis Group.

### Bilateral Pneumothorax Associated with Lung and Pleural Metastases of Breast Cancer: Report of a Case

TERUMASA KURIHARA[a,*], OSAMU KAWASHIMA[a], KEIICHI ENDO[a], YUICHI IINO[b], SUSUMU ISHIKAWA[c] and YASUO MORISHITA[c]

[a]*Department of Surgery, National Nishi-gunma Hospital, 2854 Kanai, Shibukawa, Gunma 377-8511, Japan;* [b]*Department of Emergency and Critical Care Medicine;* [c]*Second Department of Surgery, Gunma University School of Medicine, 3-39-15 Showa-machi, Maebashi, Gunma 371-8511, Japan*

*(Received 10 October 2000; In final form 29 November 2000)*

**A rare case of bilateral pneumothorax in a 54-year-old woman with advanced breast cancer associated with lung and pleural metastases is presented. The patient was admitted to our hospital complaining of unexpected severe dyspnea. A chest X-ray showed bilateral pneumothorax associated with multiple lung metastases and pleural effusions, followed by immediate pleural drainage. Although air leak and effusions of the right lung were well controlled by the conservative management, massive air leaks of the left lung had continued for 40 days. Because of patient's poor general status a surgical closure of the leaking site was selected using video-assisted thoracoscopic surgery techniques. Thoracoscopy revealed a ruptured bulla in the lower lobe ($S^6$), thus, followed by a successful bullectomy with a stapling device. We speculate that multiple pleural metastasis may disturb the normal repair mechanism of the lung tissue and cause prolonged persistent air leaks.**

*Keywords*: Breast cancer, Lung and pleural metastases, Pneumothorax, Video-assisted thoracoscopic surgery

### INTRODUCTION

Bilateral pneumothorax is a rare complication in patients with advanced breast cancer associated with lung and/or pleural metastasis. Persistent air leaks of the lung may cause severe intrathoracic infections and complications. Recently, video-assisted thoracoscopic surgery (VATS) is safely applied even in the severely ill patients [1,2]. We report a case of persistent air leak in a patient with advanced breast cancer associated with lung and pleural metastases, focusing on the etiology of persistent air leak and decision making for surgical treatment.

* Corresponding author. Tel.: +81-27-322-5901. Fax: +81-27-327-1826.

### CASE REPORT

A 54-year-old Japanese woman with advanced breast cancer was admitted to our hospital because of unexpected severe dyspnea. The patient had never consulted to hospitals, although she had noticed a mass in her left breast for 3 years. The patient was nonsmoker without a previous history of pulmonary diseases. On admission blood gas analysis at room air showed that $O_2$ saturation was 87.3%, $PO_2$ was 54.3 mmHg, $PCO_2$ was 51.7 mmHg and pH was 7.387. A chest X-ray showed bilateral pneumothorax associated with multiple lung metastases and pleural effusions (Fig. 1). The patient was immediately placed on pleural drainage. Cytologic examination of hemorrhagic pleural effusions revealed a cluster of adenocarcinoma cells, leading to a diagnosis of metastasis from breast cancer. On physical examination, a firm, fixed to the skin and chest wall, 10 × 8 cm in size mass was palpable in the left breast. Multiple daughter nodules were found in the left breast, and firm lymph nodes were palpable in the left axillas and neck. A diagnosis of advanced breast cancer (T4c N3 M1, Stage IV) was made. Pathology of biopsy specimens of the breast mass confirmed invasive ductal carcinoma (Fig. 2). Air leaks of the right lung ceased 7 days after pleural drainage. After the intrathoracic instillation of adriamycin 20 mg for two times, effusion disappeared and cytologic examination revealed no malignant cells in the right side. However, massive air leaks of the left lung had continued. Thus, we followed up the patient conservatively without intrathoracic instillation of adriamycin because of speculating that intractable air leaks might be caused by secondary pneumothorax resulted from rupture of a metastatic lung tumor. After the conservative treatment for 40 days, a thoracoscopic approach was selected to close the leaking site because of patient's poor general status.

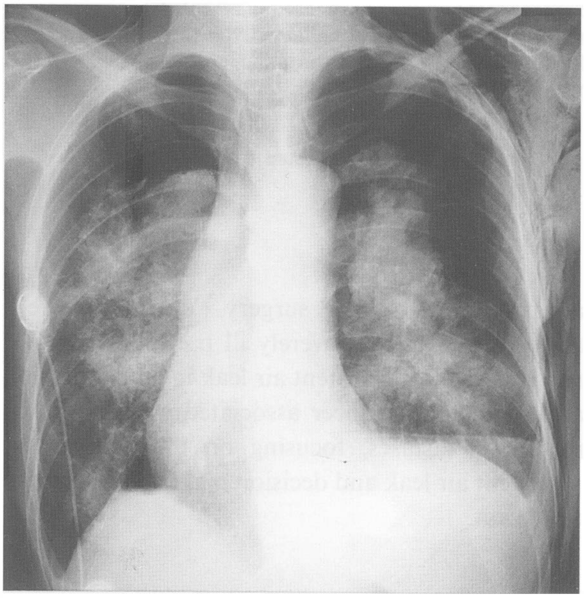

FIGURE 1 A chest X-ray on admission showing bilateral pneumothorax, multiple lung nodules, and pleural effusions.

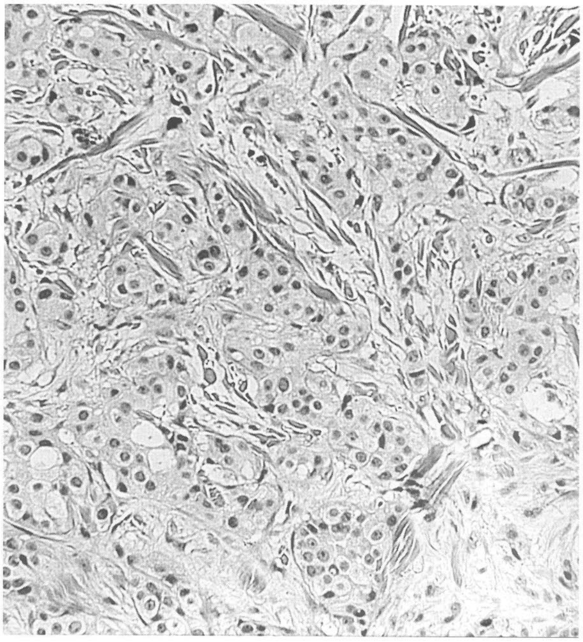

FIGURE 2 Histological findings of biopsy specimen of the breast tumor showing invasive ductal carcinoma.

Under general anesthesia, the patient was intubated with a double-lumen endotracheal tube, and was placed in a right lateral decubitus position. A thoracoscope was carefully inserted into the pleural cavity. Multiple lung metastases with

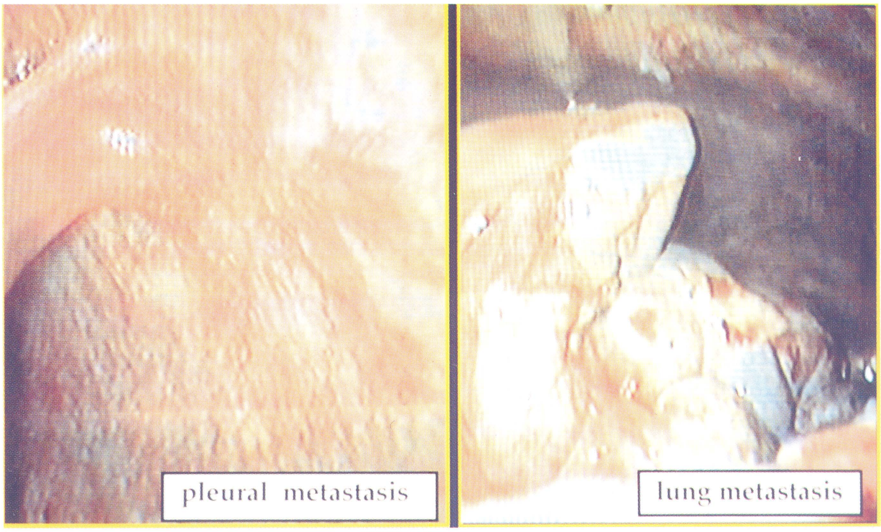

FIGURE 3 Thoracoscopic features showing wall thickening with redness of parietal pleura (left) and massive lung metastasis (right).

marked pleural indentations and wall thickening of the parietal and visceral pleura were thoracoscopically observed (Fig. 3). Within the diffuse fibrin coating over the lung, a ruptured bulla in the lower lobe ($S^6$) was identified and clarified $S^6$ bulla as the leaking site (Fig. 4). Bullectomy with a stapling device (ENDO-GIA®, United States Surgical Corp., Norwalk, CT) was successfully carried out (Fig. 4). A total operation time was 35 minutes. There were no serious complications during and after surgery.

The postoperative course was uneventful, and the drainage tube was removed three days later. Histologic findings of resected specimens showed

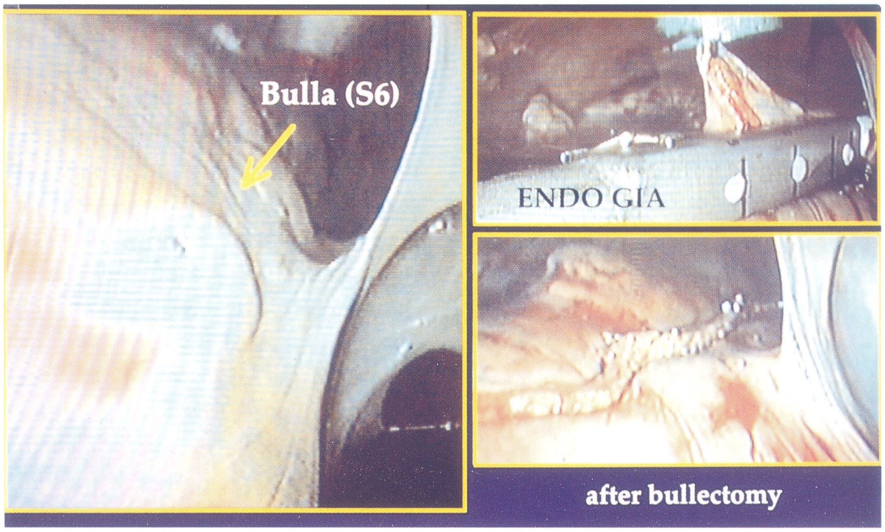

FIGURE 4 Thoracoscopic features showing a ruptured bulla in the lower lobe (left). Bullectomy with a stapling device (ENDO-GIA®) was carried out (right).

diffuse spreading of carcinoma cells within the visceral pleura. Marked invasion of cancer cells to lymphatics was also observed (Fig. 5). Adriamycin 20 mg was instilled in the left pleural cavity 2 days after surgery. Subsequently, systemic chemotherapy of CAF (cyclophosphamide 350 mg/m$^2$, adriamycin 20 mg/m$^2$, and 5-fluorouracil 350 mg/m$^2$, day 1, intravenous, every 4 weeks) and the consecutive administration of toremifene 120 mg/day were carried out. Because chemoendocrine therapy was effective, local remission and no reaccumulation of pleural effusion were certified for 6 months (Fig. 6). However, unfortunately, the patient died of progressive lung metastasis 10 months after surgery.

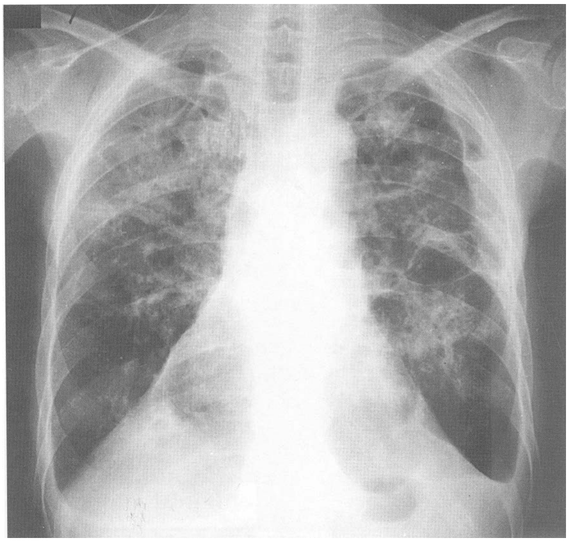

FIGURE 6 A chest X-ray after surgery and subsequent systemic chemotherapy showing disappearance of pleural effusion.

### DISCUSSION

Spontaneous pneumothorax in patients who had malignancy with lung and/or pleural metastasis is an exceptional complication. While most common primary malignancy is osteosarcoma which exhibits a rapid growth [3,4], patients with breast carcinoma rarely develop pneumothorax. Pneumothorax associated with lung metastasis is frequently provoked by treatment-induced necrosis of metastatic tumor or direct invasion of cancer to the visceral pleura [5,6]. Ruptures of antecedent bulla or blebs, check-valve mechanism due to compression of bronchiole by metastatic lesions are also noted as a cause of pneumothorax.

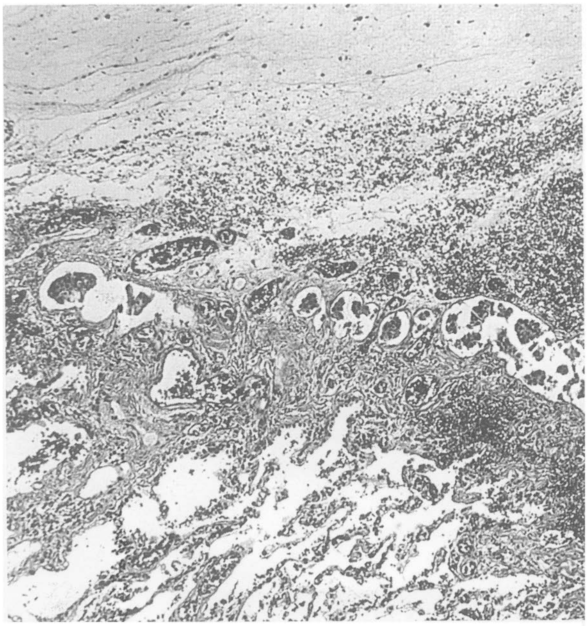

FIGURE 5 Histological findings of Resected specimen of the lung showing diffuse spreading of carcinoma cells within the visceral pleura.

In our case, we speculated secondary pneumothorax due to lung metastasis, and first selected conservative therapy. However, pneumothorax was unexpectedly caused by rupture of pre-existent bulla. Detailed thoracoscopic observation and sealing test could find out no other origin of air leak. Histologic findings of resected specimens revealed diffuse spreading of carcinoma cells within the visceral pleura and marked invasion to lymphatics. Pictures of necrosis were not clear. Pathogenesis of the left-sided pneumothorax in our case was finally considered to be ruptured bulla alone. A ruptured bulla is usually closed within 2 weeks by the normal tissue repair mechanism. However, it is quite questionable whether the normal repair mechanism can operate even when severe pleural metastasis is present. In addition, metastatic masses may disturb a sufficient pulmonary expansion. Finally, the lack of repair mechanism was speculated to be related to prolonged air leaks.

Generally, intrathoracic drainage with or without the instillation of a sclerosing agent is the management of a first choice for pneumothorax. While most patients are well controlled by this management, under some circumstances, air leaks are likely to be prolonged and required surgical closure. The timing of surgery is generally determined 14 days after intrathoracic drainage. Long-term conservative therapy has a potential to occur infection or pneumonia, sometimes leading to empyema in high-risk patients. In our case, there were some risks and an unfavorable condition in performing open thoracotomy such as high pulmonary dysfunction, association of malignant effusion, and cachectic status. Therefore, VATS technique was selected as a minimum invasive procedure. Surgical procedure was safely carried out with a smaller risk, and a total operation time was only 35 minutes. After surgery, anti-cancer therapy was safely and effectively carried out, and resulted in the improvement of her quality of life. The less invasive surgical approach using VATS may be recommended for an intractable air leaks even in patients with advanced cancer.

### *References*

[1] Jaklitsch, M.T., DeCamp, M.M., Liptay, M.J. *et al.* Video-assisted thoracic surgery in the elderly. A review of 307 cases. *Chest* 1996; **110**: 751–758.

[2] DeCamp, M.M., Jaklitsch, M.T., Mentzer, S.J. *et al.* The safety and versatility of video-thoracoscopy. A prospective analysis of 895 consecutive cases. *J. Am. Coll. Surg.* 1995; **181**: 113–120.

[3] Snevik, B. and Klepp, O. The risk of spontaneous pneumothorax in patients with osteogenic sarcoma and testicular cancer. *Cancer* 1982; **49**: 1734–1737.

[4] Dine, D.E., Cortese, D.A., Brennan, M.D. *et al.* Malignant pulmonary neoplasms predisposing to spontaneous pneumothorax. *Mayo Clin. Proc.* 1973; **48**: 541–544.

[5] Stein, M.E., Haim, N., Drumea, K. *et al.* Spontaneous pneumothorax complicating chemotherapy for metastatic seminoma. A case report and a review of the literature. *Cancer* 1995; **75**: 2710–2713.

[6] Schulman, P., Cheng, E., Cvitkovic, E. *et al.* Spontaneous pneumothorax as a result of intensive cytotoxic chemotherapy. *Chest* 1979; **75**: 194–196.

